# An Investigation of the Inverted Structure of a PBDB:T/PZT:C1-Based Polymer Solar Cell

**DOI:** 10.3390/polym15244623

**Published:** 2023-12-05

**Authors:** Tahani I. Al-Muhimeed, Shareefah Alahmari, Muhammad Ahsan, Mostafa M. Salah

**Affiliations:** 1 Department of Chemistry, College of Sciences, King Saud University, P.O. Box 2455, Riyadh 11451, Saudi Arabia; talmuhimeed@ksu.edu.sa (T.I.A.-M.); salahmariy@ksu.edu.sa (S.A.); 2 Department of Measurements and Control Systems, Silesian University of Technology, 44-100 Gliwice, Poland; muhammad.ahsan@polsl.pl; 3 Electrical Engineering Department, Future University in Egypt, Cairo 11835, Egypt

**Keywords:** all-polymer solar cell, PCE, inverted structure

## Abstract

Based on experimental results, this theoretical study presents a new approach for investigating polymers’ solar cells. P-type PZT:C1 and N-type PBDB:T were used to construct a blend for use as a photoactive layer for the proposed all-polymer solar cell. Initially, an architecture of an ITO/PEDOT:PSS/PBDB:T/PZT:C1/PFN-Br/Ag all-polymer solar device calibrated with experimental results achieved a PCE of 14.91%. A novel inverted architecture of the same solar device, proposed for the first time in this paper, achieved a superior PCE of 19.92%. Furthermore, the optimization of the doping of the transport layers is proposed in this paper. Moreover, the defect density and the thickness of the polymer are studied, and a PCE of 22.67% was achieved by the optimized cell, which is one of the highest PCEs of polymer solar devices. Finally, the optimized polymer solar cell showed good stability amidst temperature variations. This theoretical study sheds light on the inverted structure of all-polymer solar devices.

## 1. Introduction

Renewable energy resources provide promising solutions to solve the problem of increasing energy demands [1]. Several different types of solar devices have been investigated. The aim is to attain higher power conversion efficiency (PCE) values, as well as low-cost solar devices [2,3,4]. Various reports on inexpensive silicon-based structures have been published in this regard [5,6,7,8,9]. However, the reports on PCEs remain modest. New thin-film technologies have offered improved efficiency, as well as low-cost fabrication. Bulk-heterojunction, perovskites, and dye sensitized solar devices, which are types of the third generation solar devices, are currently the most extensively researched and are making the most progress. Recently, due to their lower processing costs, reasonable process flexibility, and light weight, polymer solar devices have drawn a lot of attention [10]. Additionally, because of the high material absorption coefficient of polymer solar devices, the thickness of their materials can be reduced [11].

All-polymer films of solar devices that possess a P-type integrated polymer also serve as decorative and an N-type organic materials that can be conjugated, or a small-molecule polymer accepter can be used as an absorber. Polymers, acceptors, and donors are present in all-polymer solar devices. The ongoing advancements in the development of small-molecule acceptors have caused the PCEs of polymer solar devices to reach 18% [12,13,14,15], while the efficiency values of all-polymer solar devices still fall below this PCE. The PCEs of polymer-based solar devices remain inferior to those of organic solar devices based on small molecule acceptors. This can be back to the restricted materials of promising polymer acceptors. This type of solar device offers superior qualities, including remarkable structural stability and mechanical robustness [16,17]. Numerous efforts have been undertaken to provide essential designs to produce various types of polymer solar devices.

The materials’ abbreviations and full names are mentioned in the supplementary data.

High-efficiency all-polymer solar devices were attained when mixing PBDB:T polymer donors with Y5 derivatives [18]. However, derivatives like PYT, which stem from Y5, are constrained by their elevated band gap values, which exceed 1.43 eV, producing a restricted short-circuit current density (J_sc_) [19]. Meanwhile, a PYF-T polymer acceptor with a smaller band gap value of 1.41 eV was created by Ying et al., which led to higher J_sc_ and PCE values [20]. Recently, a small-molecule acceptor polymerization strategy was introduced to improve the efficiency of all-polymer stem cells [21,22,23,24]. Based on this technology, various narrow-band-gap N-type-doped polymers have been evaluated using this approach, and PCEs higher than 13% have been documented [21,22,23,24]. At the end of 2022, based on a fused benzotriazole core, a small-molecule acceptor was polymerized using PZT-C1, which was prepared and combined with an N-type PBDB:T. The derived all-polymer solar devices obtained a record PCE of 14.9% [25].

In addition, the engineering of energy gap matching at the interfaces has demonstrated the role of the hole transfer layer (HTL) in influencing the architectures of solar devices. Numerous studies have been conducted on various HTLs in organic solar cells [26,27,28,29]. One of the most outstanding polymer compounds for usage as a HTL is PEDOT: PSS, due to its excellent conductivity, apparent transparency, and inexpensive-cost, especially for inverted solar devices [30]. PEDOT: PSS offers a synchronized work function with Indium tin oxide (ITO). As a sequence, it was used as a HTL in this study.

The process of optimizing polymer solar devices through trial-and-error experimental work can be exceedingly costly and may not guarantee success. Therefore, theoretical studies are crucial since they are more effective for either improving cell performance or understanding physics beyond the general trend of cell parameters under different settings. Numerous simulation studies have delved into polymer-based solar cells. One study revealed that a polymer solar device utilizing a PCBM/P3HT absorber film could yield an efficiency of 2.9% [31]. To determine the optimal active thickness, a simulation of the cell with the PTB7:Th/PC_71_BM mixture was carried out. The findings demonstrated an activity of 8.15% for a cell thickness of 270 nm [32]. A study found that inserting a P-type non fullerene ITIC with an N-type PBDB:T increased cell efficiency by 14.25% [33]. The simulation of a ITO/PEDOT:PSS/PT7B/PC70BM/PFN-Br/Ag device resulted in an optimization that allowed for a maximum PCE of 8% [34]. Zn(O,S) was discovered to be a matching conjugate, obtaining a PCE of 17.15%, as reported in [35], in which a PTB7/PC_70_BM blend with various electron transfer layer (ETL) materials was tested. Different simulation studies have looked at substituting PEDOT:PSS with other alternatives [36,37,38]. Up to our knowledge, there are no existing simulation studies, including the mentioned studies and others, have explored all-polymer solar devices utilizing polymerized acceptors.

This simulation study builds upon previous discussions and concentrates on enhancing the performance of polymer-based solar devices, thereby expanding their potential for indoor applications alongside conventional uses. The constructed simulation model was initially calibrated versus a fabricated solar device with the design of ITO/PEDOT:PSS/PBDB:T/PZT:C1/PFN/Ag [25]. The first design deteriorated from its comparatively low short-circuit current, which was quite strong. This simulation work presents a way to potentially enhance the efficiency of all-polymer solar devices, and this provides design guidance for future experimental investigations.

The rest of this study is organized as follows: The Section 2 (Methods and Materials) provides an overview the used simulator (SCAPS-1D) and the initial design and results of the proposed ITO/PEDOT:PSS/PBDB:T/PZT:C1/PFN-Br/Ag-based polymer solar device. The Section 3 (Results and Discussion) illustrates the advantages of the inverted architecture of the simulated device and the optimization of the main significant parameters. Moreover, the PCE’s dependence on temperature changes and the PCEs of state-of-the-art polymer solar devices are illustrated in Section 3 (Results and Discussion). Section 4 (Conclusions) concludes the study.

## 2. Methods and Materials

Solar cell capacitance simulator one dimension (SCAPS-1D) was employed for modeling and assessing the proposed solar cell. SCAPS-1D has been widely used in the simulation and modeling of thin-film solar cells [33,34,35,39]. This simulator has been calibrated in other experimental studies [33,40]. Most of the parameters of the material, like the energy gaps, electron affinities, permittivity, and mobilities, can be assigned [41]. 

This simulator solves the continuity Equations (1)–(3) to simulate solar cells. Equation (3) is the Poisson’s Equation. These equations have a self-consistent solution prior to convergence.

(1) $$\frac{1}{q}\frac{dJ_{p}}{dx}=G\left(x\right)-U$$

(2) $$\frac{1}{q}\frac{dJ_{n}}{dx}=U-G(x)$$

(3) $$\frac{d^{2}\psi }{dx^{2}}=\frac{q}{\varepsilon }(n-p+N_{A}^{-}-N_{D}^{+}+n_{t}-p_{t})$$

Table 1 summarizes the physical definitions of the parameters used in the above equations and their definitions. $G\left(x\right)$ signifies the rate of carrier generation induced by solar radiation, with *x* denoting the depth beneath the semiconductor’s exposed surface, as provided by (4):

(4) $$G\left(x\right)=\int_{0}^{\infty }G\left(\lambda ,\;x\right)d\lambda =\int_{\lambda 1}^{\lambda 2}\alpha \left(\lambda \right).(1-r\left(\lambda \right))\cdot N(\lambda ,\;0)\cdot Q(\lambda )\cdot e^{\left[-\alpha (\lambda )\;\cdot x\right]}d\lambda$$

*λ*_1_ denotes the lowest wavelength, and *λ*_2_ represents the highest wavelength range. Absorption and reflection coefficients are expressed by *α*(*λ*), and *r*(*λ*), respectively. *N*(*λ*, 0) is the illumination photons’ flux at the surface in photons.s^−1^.m^−2^, while *Q*(*λ*) is the inner QE. 

**Table 1:** The utilized parameters in SCAPS-1D.

Symbols	Definitions	Units
*U*	Recombination’s rate. The impact of ionization, deep-traps, Auger’s and radiates recombination are considered. The calculation of the recombination’s rate by deep traps in the energy gaps is carried out by Shockley-Read-Hall (SRH) theorem [42,43].	cm^−3^s^−1^
*J_p_* and *J_n_*	Hole current density and electron current density.	A/cm^2^
*ε*	Permittivity.	F/cm
*ψ*	Electrostatic potential.	V
*q*	Electron’s charge.	C
*N_D_*^+^ and *N_A_*^−^	The ionized concentrations of donors and acceptors, respectively.	cm^−3^
*n_t_* and *p_t_*	The trapped densities of electrons and holes, respectively.	cm^−3^
*n* and *p*	The concentration of electrons and holes, respectively.	cm^−3^

**Figure 1:**
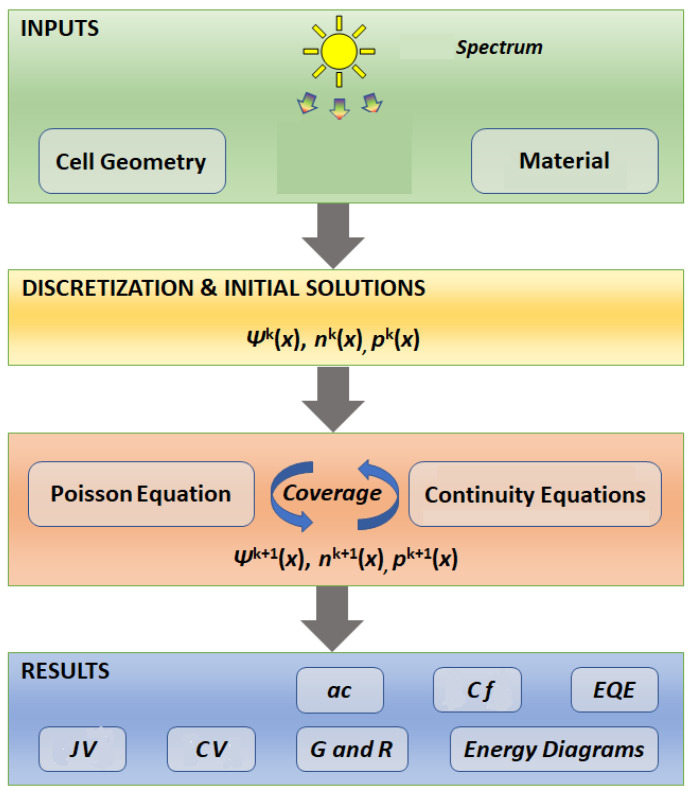
Working mechanism of SCAPS-1D.

Figure 1 illustrates the working mechanism of the simulation in SCAPS-1D. To start the simulation, firstly, insert the incident spectrum. Secondly, designing the cell geometry involves inserting the solar device’s layers, and the illumination of the top or the bottom, and the device acts as a generator or consumer. Thirdly, the materials’ parameters are assigned. Various mesh-points are utilized in the continuity equations and Poisson’s Equations (1)–(3). The finite-difference-method (FDM) is utilized to simplify the calculations. While the Scharfetter–Gummel’s method is used to model the current density [44]. Gummel’s method is used in iteration quantifying *p* and *n* in the given mesh points [45]. In all iterations, physical quantities like *G*, *U µ_p_*, and *µ_n_* are enhanced. The output characteristics that could be obtained are the external quantum efficiency (EQE), current density/voltage (JV), small signal quantities (AC capacitance/frequency (CF), capacitance/voltage (CV)), recombination rates (U), generations (G), and energy band diagrams [46]. The considered recombination mechanisms encompass Auger’s recombination, direct band-to-band recombination, and SRH theory. Additionally, characteristics related to defects, such as trap density and energy gap, could be specified within the material’s bulk or at its interfaces. While inserting defects within a bulk, the user determines the bulk minority carriers’ life times ($\tau_{n}$ and $\tau_{p}$), whereas interface defects are characterized by the recombination velocities $S_{n}$ and $S_{p}$ [47]. 

We used the mentioned models and procedure to achieve precise experimental results [33,40]. However, the disadvantage of this simulator relates to only being able to apply the analysis methods to one dimension only. As a sequence, in this simulator, the parameters’ effect in two or three dimensions cannot be studied.

**Figure 2:**
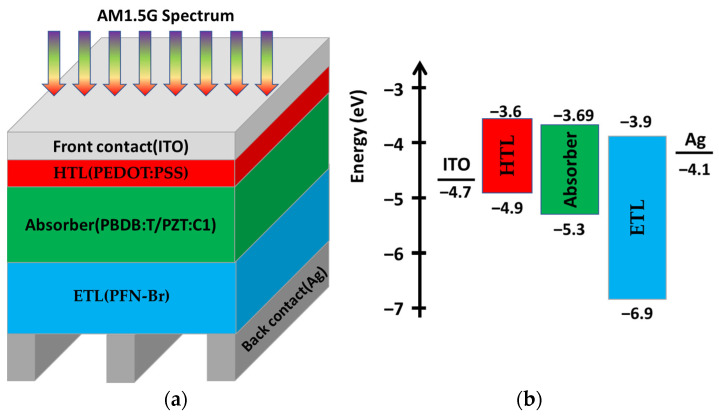
(**a**) The design of the PBDB:T/PZT:C1-based device; (**b**) energy band diagram of the used materials.

The design of the all-polymer device cell is illustrated in Figure 2a. An energy band diagram of the used materials without contacting them together illustrating the energy gaps and the affinities relative to the vacuum level is illustrated in Figure 2b. The materials we used and their functions are as follows: P-type PZT:C1 and N-type PBDB:T were used to construct a blend for use as an absorber layer sandwiched between the transport layers. A thickness of 100 nm was used for the absorber layer to obtain practical results [25]. PFN-Br was used as the ETL, while PEDOT:PSS was used as the HTL. Ag and ITO with work functions of 4.1 and 4.7 eV were used as the back and the front contacts, respectively. The used materials’ parameters are presented in Table 2. Table 3 lists the parameters of the used contacts. The boundary conditions for the contacts employed were designed to facilitate thermionics’ emissions with specific surface recombination velocities for both electrons and holes [48]. The incident spectrum for all simulations was air mass 1.5 global (AM1.5G). A room temperature of 300 k was used for all analyses. A thermal velocity of 10^7^ cms^−1^ was assumed. The hole mobility and electron mobility were extracted from the space charge limit current [25]. The density of states for the conduction and valence bands for the layers were adopted from experimental studies on polymer-based solar devices [34,49]. 

**Figure 3:**
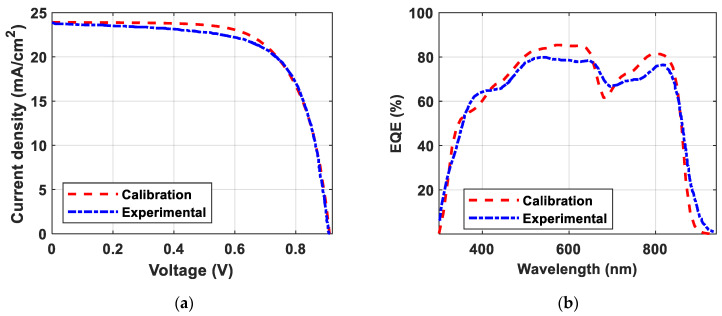
Output performance curves of the calibrated polymer solar device and the experimental cell [25]. (**a**) JV characteristics; (**b**) EQE characteristics.

The solar device’s current density/voltage (JV) curve are the most important measurable outputs of the solar cells. The JV characteristics are given by (5). Where $I_{L}$ is the light-generated current. The ideality factor is expressed by *n*, while the thermal voltage is expressed by $V_{T}$ and given by (6) [50]. Here, k represents the Boltzmann Constant, T denotes the temperature in Kelvin (K), and q stands for the electron’s charge. As a sequence, to obtain practical results from this theoretical study, we started by comparing the output results from the simulation with the experimental results. Figure 3a compares the JV curves of the experimental and calibrated solar cells, while Figure 3b compares the EQE curves. It is evident from Figure 3 that the simulation results align well with the experimental findings, with minimal differences being present, aside from the little differences in the thicknesses during the fabrication process.

(5) $$J={I_{L}-I}_{D}\left(\exp\left(\frac{V}{nV_{T}}\right)-1\right)$$

(6) $$V_{T}=\frac{kT}{q}$$

The main output metrics of the solar cells’ performance could be extracted from the JV characteristics. These metrics are the PCE, *J_sc_*, fill factor (FF), and open-circuit voltage (*V_oc_*).

The current in the solar cell does not experience a drop in voltage, and when it is short-circuited, it is called the short-circuit current. In ideal solar cells, *J_sc_* is equal to the generated light current density. As a sequence, *J_sc_* is the maximum current that could be extracted from any solar device. The current density is more commonly used than the current. The *J_sc_* is the short-circuit current divided by the area of the top of the solar device. *J_sc_* and *I_sc_* represent the largest current density and current in the solar cell, respectively.

The highest possible voltage in the solar device takes place if the net-current in it is equal to zero, which is called the open-circuit voltage. This corresponds to the forward bias in the solar device induced by the junction bias with the light-generated current. The *V_oc_* is given by (7). Consequently, the extracted power from the solar device at the *J_sc_* and the *V_oc_* is equal to zero. The maximum power point ($p_{max}$) could be calculated using (8). 

During the open-circuit and zero current density, the generated carriers recombine in the blend film. As a sequence, the recombination in the absorber can be calculated [50]. The solar device exhibits additional SRH recombination if *n* is greater than unity [51]. The values of *n* for the calibrated and experimental solar cells are 1.1 and 1.16, respectively. 

The FF is determined by the multiplication of the *J_sc_* and *V_oc_* over the multiplication of the current and voltage at the maximum power point. The sharpness of the JV curve is measured by the FF. The ideal fill factor is unity. The FF is given by (9).

The PCE is the ratio of the input power to the solar device to the output power. It is the output metric that is most used in comparing the behavior of solar devices. This efficiency varies with the input spectrum and the temperature. The solar cells’ output metrics are measured under AM1.5G and at T=300 K, as mentioned above. The PCE is given by (10).

(7) $$V_{oc}=nV_{T}\ln\frac{I_{L}}{I_{o}}$$

(8) $$p_{max}=V_{mp}I_{mp}$$

(9) $$FF=\frac{p_{max}}{V_{oc}I_{sc}\;}$$

(10) $$\eta =\frac{V_{oc}I_{sc}FF}{p_{in}}$$

Table 4 lists the main output performance parameters of the calibrated and experimental solar cells for comparison. As can be deduced from Figure 3 and Table 4, the calibrated cell can significantly simulate the experimental cell with the used material parameters and models.

## 3. Results and Discussion

**Figure 4:**
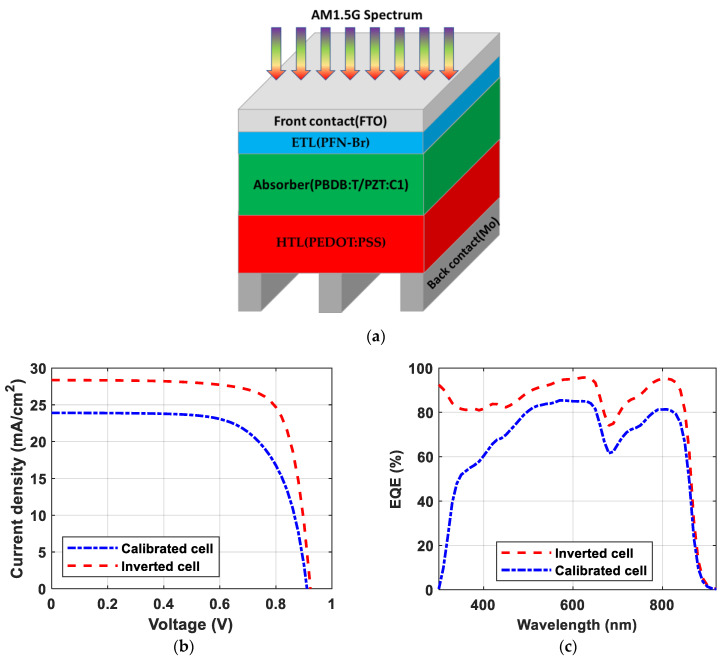
(**a**) Device design of the proposed inverted structure and traditional structure; (**b**) JV characteristics; (**c**) EQE spectra.

Firstly, the inverted architecture of the proposed cell was investigated. The front and back contacts used in the inverted structure were fluorine-doped tin oxide (FTO) and molybdenum (Mo), which had electron volt values of 4.1 eV [52], and 4.68 eV [53], respectively. The simulated inverted structure comprised FTO/ETL/absorber/HTL/Mo, as illustrated in Figure 4a. Figure 4b illustrates the JV curves of the calibrated (p-i-n) and inverted (n-i-p) structures. As can be deduced from Figure 4b, the PCE was boosted by more than 5% to be 19.92%. The FF increased to 76.09%. A current density of 28.36 mA/cm^2^ was achieved, while the *V_oc_* remained nearly consistent. The EQE of the initial and the inverted structures are illustrated in Figure 4c. This superior enhancement in the cell performance after inverting the structure is a result of illuminating the cell from the ETL side. The ETL had a lower energy gap than the HTL, allowing more incident photons into the absorber layer, resulting in an increase in the EQE. The thickness of the ETL was lower than the HTL thickness, and this could be the reason behind the decrease in the recombination rate. 

The most significant parameters of the materials used in the inverted structure design and their impact on the solar cell performance were investigated. This investigation was carried out to optimize the studied parameters in order to enhance solar cell performance.

**Figure 5:**
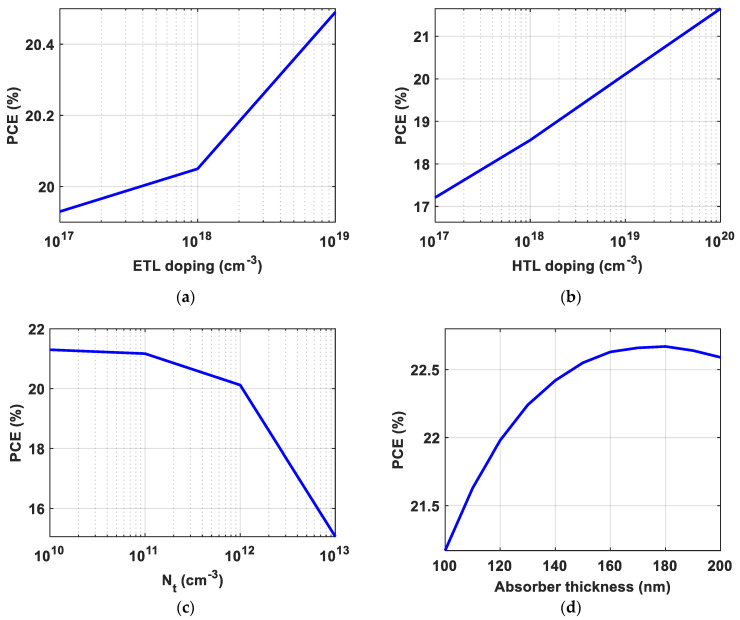

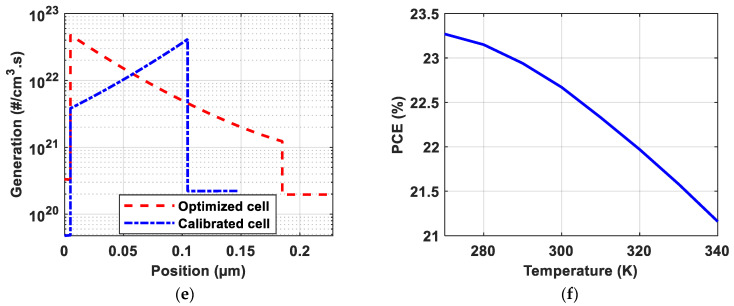
The dependence of the PCE on (**a**) ETL doping, (**b**) HTL doping, (**c**) the defect density of the absorber film, (**d**) the thickness of the polymer film, (**e**) the generation rate of the calibrated and optimized devices, and (**f**) the temperature effect on the PCE.

Doping gradation increases the carriers’ movement and the diffusion current. Consequently, the doping effect of the transport layers on the performance was investigated. Elevating the doping level of the ETL film enhances the PCE of the proposed cell. An *N_D_* of 10^18^ cm^−3^ was selected to consider practical concerns, along with a PCE of 20.05%, as presented in Figure 5a. The doping level of the HTL exerts a substantial impact on the solar device’s behavior, as illustrated in Figure 5b. An *N_A_* of 10^19^ cm^−3^ was selected, along with a PCE of 20.12%. 

The thickness of the absorber should be optimized properly to enhance the performance of the solar device. Increasing the thickness of the absorber increases the collected photons, which increases the generated carriers and enhances the PCE. However, the thickness of the absorber film and the transport film should be less than the diffusion length of the carriers to decrease the recombination rate. Consequently, the defect density in the absorber layer should be optimized before the optimization of the thickness to ensure the selection of a suitable thickness for the used diffusion length. As depicted in Figure 5c, the performance of solar cells is heavily influenced by the defect density within the absorber layer. This factor stands out as one of the most critical determinants. Lowering the defect density in the absorber film ultimately increases the PCE. However, lowering the defect density increases the cost of fabrication. As a result, an *N_t_* of 10^11^ cm^−3^ was selected to consider practical concerns, along with a high PCE of 21.17%. This enhancement is a result of changing the life time of the carriers from 1.1 × 10^4^ ns to 1.1 × 10^5^ ns when the *N_t_* decreased from 10^12^ cm^−3^ to 10^11^ cm^−3^.

Figure 5d illustrates the dependence of the PCE on the thickness of the absorber film. As can be deduced from Figure 5d, the best PCE of 22.67% was accomplished at a thickness of 180 nm, which is lower than the diffusion length (*L_n_* = 380 nm, *L_p_* = 270 nm). As expected, the generation rate increased after increasing the absorber thickness, as illustrated in Figure 5e. Figure 5f illustrates the dependence of the PCE on the variation in temperature. Figure 5f indicates that the suggested solar cell exhibits excellent stability in response to temperature fluctuations. In the range from 270 K to 340 K, the PCE decreased by about 2%.

**Figure 6:**
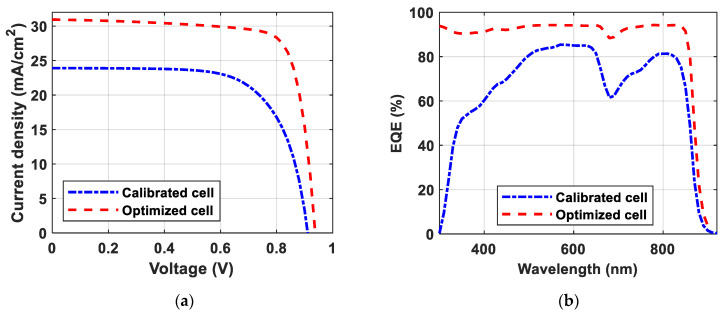
Comparison between the calibrated and optimized cells: (**a**) JV characteristics; (**b**) QE characteristics.

Figure 6a illustrates the JV characteristics of the calibrated and optimized devices. While Figure 6b illustrates the EQE characteristics.

Table 5 compares between the calibrated cell and the optimized cell to focus on the contribution of this novel work. The conclusion of this section presents a comparison between the PCE attained in this study and the PCEs of the current state-of-the-art devices, as detailed in Table 6.

## 4. Conclusions

In this theoretical study, the inverted structure of a PBDB:T/PZT:C1-based all-polymer solar device was investigated and studied utilizing SCAPS-1D. The initial design and material parameters were derived from experimental studies. The traditional structure and the inverted architecture of the ITO/HTL/absorber/ETL/Ag and FTO/ETL/absorber/HTL/Mo were investigated and compared, respectively. The inverted structure, when illuminating the cell from the ETL side, shows a superior performance in comparison to the traditional structure. The PCE of the inverted structure is 19.92%, while the PCE of the traditional structure is 14.91%. This enhancement in PCE is a result of increasing the J_SC_ from 23.9 mA/cm^2^ to 28.36 mA/cm^2^ and the FF from 68.45% to 76.09%. Consequently, it can be deduced from this study that illuminating the solar cell from the ETL side is recommended over doing so from the HTL side because the ETL has a wider energy gap and is less thick than the HTL. The impacts of several parameters were studied. The doping concentrations of the transport layers should be properly selected to improve the collection efficiency of the generated carriers. The behavior of the solar device is significantly affected by the presence of bulk defects in the absorber layer. However, decreasing the defects necessitates higher costs in the fabrication process. When the defect density was 10^11^ cm^−3^ with a carrier lifetime of 1.1 × 10^5^ ns, the PCE was 21.17%. Moreover, the thickness of the absorber film was optimized by increasing it to collect more photons, but its thickness was lower than the diffusion length. As a consequence, a PCE of 22.67% can be obtained when the absorber thickness is 180 nm. Finally, the proposed polymer solar device with the inverted structure showed good stability amidst temperature variations. The PCE decreased from 23.27% to 21.16% in response to a 70 K temperature increase. This theoretical study opens the door for achieving high PCEs via the design of polymer-based solar devices.

## Figures and Tables

**Table 2:** Materials’ parameters of the proposed polymer-based solar device [25,34,49].

Parameters	HTL (PEDOT:PSS)	Absorber (PBDB:T/PZT:C1)	ETL (PFN-Br)
Energy gap (eV)	1.3	1.61	3
Thickness (nm)	43	100	5
Affinity (eV)	3.6	3.69	3.9
Relative permittivity	3.5		
${µ}_{n}$ (cm^2^V^−1^s^−1^)	8.0 × 10^−4^	5.13 × 10^−4^	1.0 × 10^−4^
${µ}_{p}$ (cm^2^V^−1^s^−1^)		2.53 × 10^−4^	1.0 × 10^−6^
Conduction and valence bands’ density of states (cm^−3^)	1 × 10^21^		
*N_A_* (cm^−3^)	1 × 10^19^	-	-

**Table 3:** The solar cell’s contact parameters [48].

Contact		Work Functions (eV)	Holes’ Surface Recombination Velocity (cms^−1^)	Electrons’ Surface Recombination Velocity (cms^−1^)
Back contact	Ag	4.1	10^7^	10^5^
Front contact	ITO	4.7	10^5^	10^7^

**Table 4:** Main output metrics parameters of the calibrated polymer solar device and the experimental cell.

	PCE [%]	*V_OC_* [V]	*J_SC_* [mA/cm^2^]	FF [%]
Experimental [25]	14.90	0.912	23.90	68.50
Calibration	14.91	0.912	23.90	68.45

**Table 5:** Solar cell key metrics of the calibrated and optimized cells.

	PCE [%]	*V_OC_* [V]	*J_SC_* [mA/cm^2^]	FF [%]
Calibrated Cell	14.91	0.912	23.90	68.45
Optimized Cell	22.67	0.938	30.94	78.08

**Table 6:** A comparison between the PCE achieved in this work and the PCEs of state-of-the-art devices.

ETL	HTL	Absorber	PCE (%)	Type	Reference
PFN-Br	PEDOT:PSS	PM6/Y6	15.79	Ex.	[14]
PFN-Br		PBDB-T/PZT-γ	15.80		[24]
PFN		PBDB:T/PZT:C1	14.90		[25]
PDINN		PBDB-T/PN-Se	16.16		[54]
PDINN		PM6/PY-IT	16.41		[55]
ZrAcAc		PY-IT:BNT/PM6	16.09		[56]
PDIN		PY2F-T/PYT/PM6	17.2		[57]
PDIN		D18/N3	18.56		[58]
PFN-Br		PTB7/PC_70_BM	8.18	Sim.	[34]
Zn(O,S)		PTB7/PC_70_BM	17.15		[35]
PFN	CuI	PBDB:T/PZT:C1	20.87		[59]
PFN	PEDOT:PSS	PBDB:T/PZT:C1	22.67	Sim. Inverted structure	This study

Ex. = experiment; Sim. = simulation.

## Data Availability

Data are contained within the article.

## Supplementary Materials

The following supporting information can be downloaded at: https://www.mdpi.com/article/10.3390/polym15244623/s1.

## References

[1] Shaheen M.A.M., Hasanien H.M., Turky R.A., Ćalasan M., Zobaa A.F., Aleem S.H.E.A. (2021). OPF of Modern Power Systems Comprising Renewable Energy Sources Using Improved CHGS Optimization Algorithm. Energies.

[2] Petrović-Ranđelović M., Kocić N., Stojanović-Ranđelović B. (2020). The importance of renewable energy sources for sustainable development. Econ. Sustain. Dev..

[3] Green M., Dunlop E., Hohl-Ebinger J., Yoshita M., Kopidakis N., Hao X. (2021). Solar cell efficiency tables (version 57). Prog. Photovolt. Res. Appl..

[4] Okil M., Salem M.S., Abdolkader T.M., Shaker A. (2021). From Crystalline to Low-cost Silicon-based Solar Cells: A Review. Silicon.

[5] Putnam M.C., Boettcher S.W., Kelzenberg M.D., Turner-Evans D.B., Spurgeon J.M., Warren E.L., Briggs R.M., Lewis N.S., Atwater H.A. (2010). Si microwire-array solar cells. Energy Environ. Sci..

[6] Salem M.S., Zekry A., Shaker A., Abouelatta M. Design and simulation of proposed low cost solar cell structures based on heavily doped silicon wafers. Proceedings of the 2016 IEEE 43rd Photovoltaic Specialists Conference (PVSC).

[7] Salem M.S., Alzahrani A.J., Ramadan R.A., Alanazi A., Shaker A., Abouelatta M., Gontrand C., Elbanna M., Zekry A. (2020). Physically Based Analytical Model of Heavily Doped Silicon Wafers Based Proposed Solar Cell Microstructure. IEEE Access.

[8] Salem M., Zekry A., Abouelatta M., Alshammari M.T., Alanazi A., Al-Dhlan K.A., Shaker A. (2021). Influence of base doping level on the npn microstructure solar cell performance: A TCAD study. Opt. Mater..

[9] Kim G., Lim J.W., Kim J., Yun S.J., Park M.A. (2020). Transparent Thin-Film Silicon Solar Cells for Indoor Light Harvesting with Conversion Efficiencies of 36% without Photodegradation. ACS Appl. Mater. Interfaces.

[10] Gnida P., Amin M.F., Pająk A.K., Jarząbek B. (2022). Polymers in High-Efficiency Solar Cells: The Latest Reports. Polymers.

[11] Khanam J.J., Foo S.Y. (2019). Modeling of High-Efficiency Multi-Junction Polymer and Hybrid Solar Cells to Absorb Infrared Light. Polymers.

[12] Meng L., Zhang Y., Wan X., Li C., Zhang X., Wang Y., Ke X., Xiao Z., Ding L., Xia R. (2018). Organic and solution-processed tandem solar cells with 17.3% efficiency. Science.

[13] Yuan J., Zhang Y., Zhou L., Zhang G., Yip H.-L., Lau T.-K., Lu X., Zhu C., Peng H., Johnson P.A. (2019). Single-Junction Organic Solar Cell with over 15% Efficiency Using Fused-Ring Acceptor with Electron-Deficient Core. Joule.

[14] Li S., Zhan L., Jin Y., Zhou G., Lau T., Qin R., Shi M., Li C., Zhu H., Lu X. (2020). Asymmetric Electron Acceptors for High-Efficiency and Low-Energy-Loss Organic Photovoltaics. Adv. Mater..

[15] Best Research-Cell Efficiency Chart|Photovoltaic Research|NREL. https://www.nrel.gov/pv/cell-efficiency.html.

[16] Lee C., Lee S., Kim G.U., Lee W., Kim B.J. (2019). Recent Advances, Design Guidelines, and Prospects of All-Polymer Solar Cells. Chem. Rev..

[17] Xu Y., Yuan J., Zhou S., Seifrid M., Ying L., Li B., Huang F., Bazan G.C., Ma W. (2019). Ambient Processable and Stable All-Polymer Organic Solar Cells. Adv. Funct. Mater..

[18] Luo Z., Liu T., Ma R., Xiao Y., Zhan L., Zhang G., Sun H., Ni F., Chai G., Wang J. (2020). Precisely Controlling the Position of Bromine on the End Group Enables Well-Regular Polymer Acceptors for All-Polymer Solar Cells with Efficiencies over 15%. Adv. Mater..

[19] Wu Q., Wang W., Wang T., Sun R., Guo J., Wu Y., Jiao X., Brabec C.J., Li Y., Min J. (2020). High-performance all-polymer solar cells with only 0.47 eV energy loss. Sci. China Chem..

[20] Yu H., Qi Z., Yu J., Xiao Y., Sun R., Luo Z., Cheung A.M.H., Zhang J., Sun H., Zhou W. (2020). Fluorinated End Group Enables High-Performance All-Polymer Solar Cells with Near-Infrared Absorption and Enhanced Device Efficiency over 14%. Adv. Energy Mater..

[21] Peng F., An K., Zhong W., Li Z., Ying L., Li N., Huang Z., Zhu C., Fan B., Huang F. (2020). A Universal Fluorinated Polymer Acceptor Enables All-Polymer Solar Cells with >15% Efficiency. ACS Energy Lett..

[22] Wang W., Wu Q., Sun R., Guo J., Wu Y., Shi M., Yang W., Li H., Min J. (2020). Controlling Molecular Mass of Low-Band-Gap Polymer Acceptors for High-Performance All-Polymer Solar Cells. Joule.

[23] Jia T., Zhang J., Zhong W., Liang Y., Zhang K., Dong S., Ying L., Liu F., Wang X., Huang F. (2020). 14.4% efficiency all-polymer solar cell with broad absorption and low energy loss enabled by a novel polymer acceptor. Nano Energy.

[24] Fu H., Li Y., Yu J., Wu Z., Fan Q., Lin F., Woo H.Y., Gao F., Zhu Z., Jen A.K.-Y. (2021). High Efficiency (15.8%) All-Polymer Solar Cells Enabled by a Regioregular Narrow Bandgap Polymer Acceptor. J. Am. Chem. Soc..

[25] Fu H., Li Y., Wu Z., Lin F.R., Woo H.Y., Jen A.K. (2022). Side-Chain Substituents on Benzotriazole-Based Polymer Acceptors Affecting the Performance of All-Polymer Solar Cells. Macromol. Rapid Commun..

[26] Anrango-Camacho C., Pavón-Ipiales K., Frontana-Uribe B.A., Palma-Cando A. (2022). Recent Advances in Hole-Transporting Layers for Organic Solar Cells. Nanomaterials.

[27] Xu H., Yuan F., Zhou D., Liao X., Chen L., Chen Y. (2020). Hole transport layers for organic solar cells: Recent progress and prospects. J. Mater. Chem. A.

[28] Ogundele A.K., Mola G.T. (2022). Ternary atoms alloy quantum dot assisted hole transport in thin film polymer solar cells. J. Phys. Chem. Solids.

[29] Zhong Y., Li Y., Lan X., Wang J., Wang J., Zhang Y. (2021). Enhancing Efficiency and Stability of Polymer Solar Cells Based on CuI Nanoparticles as the Hole Transport Layer. IEEE J. Photovoltaics.

[30] Wang Z., Zou X., Zhao M., Wang J., Wang X., Hao Y., Wang H. (2022). A simple doping strategy to improve PEDOT:PSS charge extraction capability in polymer solar cells. Sol. Energy.

[31] Islam S. (2017). Analytical modeling of organic solar cells including monomolecular recombination and carrier generation calculated by optical transfer matrix method. Org. Electron..

[32] Zang Y., Xin Q., Zhao J., Lin J. (2018). Effect of Active Layer Thickness on the Performance of Polymer Solar Cells Based on a Highly Efficient Donor Material of PTB7-Th. J. Phys. Chem. C.

[33] Abdelaziz W., Shaker A., Abouelatta M., Zekry A. (2019). Possible efficiency boosting of non-fullerene acceptor solar cell using device simulation. Opt. Mater..

[34] Alahmadi A.N.M. (2022). Design of an Efficient PTB7:PC70BM-Based Polymer Solar Cell for 8% Efficiency. Polymers.

[35] Moiz S.A., Alzahrani M.S., Alahmadi A.N.M. (2022). Electron Transport Layer Optimization for Efficient PTB7:PC_70_BM Bulk-Heterojunction Solar Cells. Polymers.

[36] Widianto E., Firdaus Y., Shobih, Pranoto L.M., Triyana K., Santoso I., Nursam N.M. (2022). Device modeling of two-dimensional hole transport materials for boosting the performance of non-fullerene acceptor bulk heterojunction organic solar cells. Opt. Mater..

[37] Sharma B., Mathur A., Rajput V., Singh I., Singh B. (2021). Device modeling of non-fullerene organic solar cell by incorporating CuSCN as a hole transport layer using SCAPS. Optik.

[38] Nowsherwan G.A., Samad A., Iqbal M.A., Mushtaq T., Hussain A., Malik M., Haider S., Pham P.V., Choi J.R. (2022). Performance Analysis and Optimization of a PBDB-T:ITIC Based Organic Solar Cell Using Graphene Oxide as the Hole Transport Layer. Nanomaterials.

[39] Abdelaziz W., Zekry A., Shaker A., Abouelatta M. (2020). Numerical study of organic graded bulk heterojunction solar cell using SCAPS simulation. Sol. Energy.

[40] Salah M.M., Abouelatta M., Shaker A., Hassan K.M., Saeed A. (2019). A comprehensive simulation study of hybrid halide perovskite solar cell with copper oxide as HTM. Semicond. Sci. Technol..

[41] Burgelman M., Decock K., Niemegeers A., Verschraegen J., Degrave S. (2016). SCAPS Manual.

[42] Shockley W., Read W.T. (1952). Statistics of the Recombinations of Holes and Electrons. Phys. Rev. B.

[43] Hall R.N. (1952). Electron-Hole Recombination in Germanium. Phys. Rev. B.

[44] Scharfetter D., Gummel H. (1969). Large-signal analysis of a silicon Read diode oscillator. IEEE Trans. Electron Devices.

[45] Gummel H. (1964). A self-consistent iterative scheme for one-dimensional steady state transistor calculations. IEEE Trans. Electron Devices.

[46] Boumaour M., Sali S., Kermadi S., Zougar L., Bahfir A., Chaieb Z. (2019). High efficiency silicon solar cells with back ZnTe layer hosting IPV effect: A numerical case study. J. Taibah Univ. Sci..

[47] Basyoni M.S.S., Salah M.M., Mousa M., Shaker A., Zekry A., Abouelatta M., Alshammari M.T., Al-Dhlan K.A., Gontrand C. (2021). On the Investigation of Interface Defects of Solar Cells: Lead-Based vs Lead-Free Perovskite. IEEE Access.

[48] Hussain S.S., Riaz S., Nowsherwan G.A., Jahangir K., Raza A., Iqbal M.J., Sadiq I., Naseem S. (2021). Numerical Modeling and Optimization of Lead-Free Hybrid Double Perovskite Solar Cell by Using SCAPS-1D. J. Renew. Energy.

[49] Aeberhard U., Schiller A., Masson Y., Zeder S.J., Blülle B., Ruhstaller B. (2022). Analysis and Optimization of Organic Tandem Solar Cells by Full Opto-Electronic Simulation. Front. Photon.

[50] Salem M.S., Shaker A., Abouelatta M., Alanazi A., Al-Dhlan K.A., Almurayziq T.S. (2022). Numerical analysis of hole transport layer-free antimony selenide solar cells: Possible routes for efficiency promotion. Opt. Mater..

[51] Leong W.L., Ooi Z.-E., Sabba D., Yi C., Zakeeruddin S.M., Graetzel M., Gordon J.M., Katz E.A., Mathews N. (2016). Identifying Fundamental Limitations in Halide Perovskite Solar Cells. Adv. Mater..

[52] Das A., Peu S.D., Akanda A.M., Salah M.M., Hossain S., Das B.K. (2023). Numerical Simulation and Optimization of Inorganic Lead-Free Cs_3_Bi_2_I_9_-Based Perovskite Photovoltaic Cell: Impact of Various Design Parameters. Energies.

[53] Wang D., Wu J., Guo H., Wu M., Wu L., Zhang S., Ao J., Wang H., Zhang Y. (2020). Tuning the Work Function of the Metal Back Contact toward Efficient Cu _2_ ZnSnSe _4_ Solar Cells. Sol. RRL.

[54] Du J., Hu K., Zhang J., Meng L., Yue J., Angunawela I., Yan H., Qin S., Kong X., Zhang Z. (2021). Polymerized small molecular acceptor based all-polymer solar cells with an efficiency of 16.16% via tuning polymer blend morphology by molecular design. Nat. Commun..

[55] Zhang G., Wang L., Zhao C., Wang Y., Hu R., Che J., He S., Chen W., Cao L., Luo Z. (2022). Efficient All-Polymer Solar Cells Enabled by Interface Engineering. Polymers.

[56] Ji X., Xiao Z., Sun H., Guo X., Ding L. (2021). Polymer acceptors for all-polymer solar cells. J. Semicond..

[57] Wu B., Yin B., Duan C., Ding L. (2021). All-polymer solar cells. J. Semicond..

[58] Jin K., Xiao Z., Ding L. (2021). D18, an eximious solar polymer!. J. Semicond..

[59] Salem M.S., Shaker A., Salah M.M. (2023). Device Modeling of Efficient PBDB-T:PZT-Based All-Polymer Solar Cell: Role of Band Alignment. Polymers.

