# Supplementary material for: An Investigation of the Inverted Structure of a PBDB:T/PZT:C1-Based Polymer Solar Cell
Source: Polymers (Basel). 2023 Dec 5;15(24):4623. doi: 10.3390/polym15244623 (PMC10747590; doi:10.3390/polym15244623)
Supplement: Supplementary file 1 [file polymers-15-04623-s001.zip › polymers-2689319-SI.pdf]

polymers

MDPI

Supplementary Data

Article

An Investigation of the Inverted Structure of A PBDB:T/PZT:C1-Based Polymer Solar Cell

Tahani I. Al-Muhimeed [1], Shareefah Alahmari [1], Muhammad Ahsan [2] and Mostafa M. Salah [3,*]

[1] Department of Chemistry, College of Sciences, King Saud University, P.O. Box 2455, Riyadh 11451, Saudi Arabia; talmuhimeed@ksu.edu.sa (T.I.A.-M.); salahmariy@ksu.edu.sa (S.A.)
[2] Department of Measurements and Control Systems, Silesian University of Technology, 44-100 Gliwice, Poland; muhammad.ahsan@polsl.pl
[3] Electrical Engineering Department, Future University in Egypt, Cairo 11835, Egypt
* Correspondence: mostafa.abdulkhalek@fue.edu.eg

Materials' Names.

Abbreviation	Full-Names
PBDB-T	Poly[(2,6-(4,8-bis(5-(2-ethylhexyl)thiophen-2-yl)-benzo[1,2-b:4,5-b']dithiophene))-alt-(5,5-(1',3'-di-2-thienyl-5',7'-bis(2-ethylhexyl)benzo[1',2'-c:4',5'-c']dithiophene-4,8-dione)]
PZT	lead zirconate titanate (Pb[Zr(x)Ti(1-x)]O3)
ITO	Indium tin oxide
PEDOT:PSS	poly(3,4-ethylenedioxythiophene) polystyrene sulfonate
PFN-Br	Poly(9,9-bis(3'-(N,N-dimethyl)-N-ethylammoinium-propyl-2,7-fluorene)-alt-2,7-(9,9-dioctylfluorene))dibromide
CuI	Copper Iodide
P3HT	Regioregular poly(3-hexylthiophene)
PCBM	fullerene derivative [6,6]-phenyl-C_{61}-butyric acid methyl ester
Zn(O,S)	Zinc oxysulphide
Ag	Silver
PM6	Poly[(2,6-(4,8-bis(5-(2-ethylhexyl-3-fluoro)thiophen-2-yl)-benzo[1,2-b:4,5-b']dithiophene))-alt-(5,5-(1',3'-di-2-thienyl-5',7'-bis(2-ethylhexyl)benzo[1',2'-c:4',5'-c']dithiophene-4,8-dione)]
Y6	thienothienopyrrolo-thienothienoindole (TTP-TTI) core base and 2-(5,6-difluoro-3-oxo-2,3-dihydro-1H-inden-1-ylidene)malononitrile (2FIC)
PN-Se	Polyphosphazenes-selenium
PDINN	N,N'-Bis{3-[3-(Dimethylamino)propylamino]propyl}perylene-3,4,9,10-tetracarboxylic diimide
PY	Pyridine
PTB7	Poly [[4,8-bis[(2-ethylhexyl)oxy]benzo[1,2-b:4,5-b']dithiophene-2,6-diyl][3-fluoro-2-[(2-ethylhexyl)carbonyl]thieno[3,4-b]thiophenediyl]]
ZrAcAc	zirconium acetylacetonate
D18	Poly[(2,6-(4,8-bis(5-(2-ethylhexyl-3-fluoro)thiophen-2-yl)-benzo[1,2-b:4,5-b']dithiophene))-alt-5,5'-(5,8-bis(4-(2-butyloctyl)thiophen-2-yl)dithieno[3',2':3,4;2'',3'':5,6]benzo[1,2-c][1,2,5]thiadiazole)]
N3	azide Azide ion Hydrazoate Azide anion
